# Draft Genome Sequence of Neurospora crassa Strain FGSC 73

**DOI:** 10.1128/genomeA.00074-15

**Published:** 2015-04-02

**Authors:** Scott E. Baker, Wendy Schackwitz, Anna Lipzen, Joel Martin, Sajeet Haridas, Kurt LaButti, Igor V. Grigoriev, Blake A. Simmons, Kevin McCluskey

**Affiliations:** a DOE Joint BioEnergy Institute, Emeryville, California, USA; b Environmental Molecular Sciences Laboratory, Pacific Northwest National Laboratory, Richland, Washington, USA; c DOE Joint Genome Institute, Walnut Creek, California, USA; d Sandia National Laboratories, Livermore, California, USA; e Fungal Genetics Stock Center, Kansas State University, Manhattan, Kansas, USA

## Abstract

We report the elucidation of the complete genome of the Neurospora crassa (Shear and Dodge) strain FGSC 73, a *mat-a*, *trp-3* mutant strain. The genome sequence around the idiotypic mating type locus represents the only publicly available sequence for a *mat-a* strain. 40.42 Megabases are assembled into 358 scaffolds carrying 11,978 gene models.

## GENOME ANNOUNCEMENT

Neurospora crassa is a well-established model organism and was the first filamentous fungus to have a publicly available genome sequence (1). It is best known as the organism utilized by Beadle and Tatum to establish the “one gene, one enzyme” hypothesis (2), and it has been utilized recently for studies of cell biology, gene regulation, genome defense, population genetics, and genomics (3). The earliest published report of a gene mutation leading to the loss of an essential enzymatic activity in N. crassa was for the synthesis of pyridoxine in Neurospora (then called *Sitophila*) (2). Mutation surveys were carried out, leading to the first description of tryptophan requiring mutants in 1944 (4) and over 50 “tryptophanless” strains reported in 1950 (5). The Fungal Genetics Stock Center is the repository for 20,178 N. crassa genetic mutant strains, of which 6,630 are classical mutants. FGSC 73 has the C83 (td1) allele of *trp-3* in a mixed genetic background and is one of 80 strains carrying 40 unique *trp-3* alleles. The C83 allele was the first trp-3 allele described (6) and was generated by UV irradiation. The high-resolution genetic map and broad collection of classical mutants makes N. crassa an ideal organism for next-generation sequencing approaches aimed at linking phenotype with genotype (7–9).

We have performed whole-genome shotgun sequencing to 144.8× coverage of FGSC 73 using the Illumina sequencing platform. The genome sequence was assembled using AllPathsLG version R42328. The size of the assembled genome is 40.42 Mb and comprises 757 contigs and 358 scaffolds (316 are at least 2 kbp in length). Using the JGI annotation pipeline (10), 11,978 gene models were generated. This is in contrast to 10,357 gene models currently associated with the most recent annotation of N. crassa version 12 generated at the Broad Institute. The FGSC 73 genome contained 20,779 single-nucleotide polymorphisms (SNPs) and 2,485 insertion/deletions (indels). The amount of SNPs and indels are consistent with previously published resequencing studies (7, 8), and with this strain having been developed before the research community adopted a shared genetic lineage.

As part of a targeted sequencing project, the *trp-3* locus of this *mat-a* strain has been sequenced previously (11) and the identification of the mutation at the trp-3 locus as a deletion of one adenosine at position 1021 causing a truncation of the predicted protein at residue 379 is recapitulated in the present study.

### Nucleotide sequence accession numbers.

This whole-genome shotgun project has been deposited in DDBJ/ENA/GenBank under the accession number JTEW00000000. The version described in this paper is the first version, JTEW00000000.1.
